# Editorial: Case reports in cardiac rhythmology: 2022

**DOI:** 10.3389/fcvm.2023.1276721

**Published:** 2023-08-29

**Authors:** Andrea Ballatore, Alexander H. Maass, Giovanni Peretto, Elsayed Z. Soliman, Masateru Takigawa, Matteo Anselmino

**Affiliations:** ^1^ Division of Cardiology, Cardiovascular and Thoracic Department, “Citta della Salute e della Scienza” Hospital, Turin, Italy; ^2^ Department of Medical Sciences, University of Turin, Turin, Italy; ^3^ Department of Cardiology, University Medical Center Groningen, University of Groningen, Groningen, Netherlands; ^4^ Department of Cardiac Electrophysiology and Arrhythmology, IRCCS San Raffaele Scientific Institute, Milan, Italy; ^5^ Section on Cardiovascular Medicine, Epidemiological Cardiology Research Center, Wake Forest School of Medicine, Winston Salem, NC, United States; ^6^ Division of Advanced Arrhythmia Research, Department of Cardiovascular Medicine, Tokyo Medical and Dental University, Tokyo, Japan

**Keywords:** electrophysiology, case reports, cardiac arrhythmias, cardiac pacing, catheter ablation

**Editorial on the Research Topic** Case reports in cardiac rhythmology: 2022

Medical science is characterized by profound interactions among different disciplines, with each discipline impacting the other in different ways. Cardiac electrophysiology is no exception, as several alterations in the cardiac rhythm can be secondary to medical disorders otherwise unrelated to the development of arrhythmias. In the present volume of the *Journal*, three great examples of these interactions are reported: Omer et al. described a case in which initiation of lamotrigine in a patient with history of epilepsy lead to the unmasking of a type 1 Brugada pattern, the development of arrhythmic syncopal events, and the induction of ventricular fibrillation during ventricular programmed electrical stimulation. Duon-Quy et al. reported the use of plasmapheresis for the treatment of heart block secondary to Guillain-Barré syndrome elicited by COVID-19 infection. Finally, Duan et al. showed a case in which a carotid body tumor resection was complicated by 40 s of asystole and cardiac arrest due to carotid sinus hypersensitivity highlighting the interplay between the heart and the autonomic nervous system. In addition to the connection between systemic disease and the heart, specific cardiac pathologies can also impact electrophysiological properties. For example, coronary artery disease is frequently associated with both brady- and tachy-arrhythmias. Development of heart block in the acute phases of myocardial infarction is a common complication; however, Zhang et al. and Wu et al. described two peculiar cases in which myocardial infarction was associated with pacemaker loss of capture, pointing out a new possible mechanism of pacemaker dysfunction.

In recent years, cardiac electrophysiology has undergone significant transformation and witnessed remarkable advancements in technologies and instruments, proving to be highly efficient and helpful in several occasions as described by Li et al. Nevertheless, critical reasoning and maintaining a logical approach to electrical signals continue to play a fundamental role, and despite the advancements, the “traditional” 12-lead electrocardiography has stood the test of time, persisting as the initial and often adequate step in diagnosing cardiac arrhythmias. Ren et al. described a case of dual atrioventricular nodal non-reentrant tachycardia, an easily missed arrhythmia with potential deleterious effects, that, however, can be “simply” diagnosed on surface ECG. On the other hand, an “old” technique can benefit from renowned instruments, as demonstrated by the report of Hawryszko et al. in which a common smartwatch with the possibility to record a single lead ECG helped in the diagnosis of atrioventricular nodal reentrant tachycardia in a pregnant woman. In this delicate scenario, the use of a non-invasive technique helped in ruling out more severe cardiac arrhythmias leading to the correct management of the case. Use of wearable devices has grown exponentially in recent years and physicians will have to face increasing demand for the interpretation of a significant amount of data (1, 2). The clinical implications, as suggested the results of the LOOP study for the screening of atrial fibrillation (3), still need to be clarified. These reports, moreover, highlight recent and renewed attention to the AV node physiology and anatomy (4), an extremely complex structure, as originally claimed by the seminal work of Tawara. Similarly, in WPW cases, precise knowledge of the ECG and the correlation between ECG waveforms and cardiac anatomy are necessary to appropriately plan the ablation procedure. Yang et al. described a brilliant example in which correct ECG interpretation, at baseline and during programmed stimulation, led to the prompt identification of an atypical bypass tract. Similarly, Zhao et al. reported a case in which surface ECG helped in raising the suspicion of dextrocardia and finally guided the identification and ablation of an atrioventricular accessory pathway in this peculiar scenario.

Evidence-based approach in Medicine is founded on the results of properly conducted randomized controlled trials, with a large number of patients to guarantee the necessary statistical power to confirm, or reject, a thorough scientific hypothesis. However, cardiac electrophysiology has traditionally grown on the observation of an event in a small number of cases, providing insights into the mechanism underlying a specific phenomenon. The cornerstone of atrial fibrillation ablation is currently obtaining pulmonary vein isolation by means of different sources of energy, on the basis of the landmark study by Haissaguerre et al. (5); nevertheless, atrial fibrillation is a complex arrhythmia and further lesion sets may be warranted both to modify the substrate that sustains the arrhythmia (6) than to eliminate extra pulmonary vein triggers, as shown by Tao et al.

The anatomical substrate is the determinant of the development of arrhythmias and of their characteristics, therefore, precise knowledge of the anatomy is crucial for understanding and correctly treating arrhythmias, as in the case reported by Raina et al. Moreover, correct knowledge of cardiac anatomy is fundamental in preventing the occurrence of complications. New tools such as 3D printing and creation of accurate anatomical models can help with training, learning new techniques, and planning for a complex procedure (Wei et al.). Complications are intrinsically related to invasive procedures and, at times, are unpredictable and almost inevitable (De Innocentiis et al., Huang et al., Kim et al., Lo and Chen, Sha et al.); however, accurate knowledge of the anatomical relationship and caution during the planning of the procedure may surely help to reduce the possibility of severe complications.

“*Doctors without [knowledge of] anatomy are like moles. They work in the dark and the work of their hands are mounds*.” Tiedemann, Heidelberg, 1781–1861.
